# Cold and Wet a Cause of Camp Disease, and Its Modus Operandi

**Published:** 1863

**Authors:** C. A. Hunt

**Affiliations:** Surgeon of 126th Reg’t Ill. Vols.


ARTICLE XIV.

### COLD AND WET A CAUSE OF CAMP DISEASE, AND ITS MODUS OPERANDI.

By C. A. HUNT, M.D., Surgeon of 126th Reg't Ill. Vol.

My sanitary report of March, having been suggested by the Medical Director for the consideration of the Society, and myself being appointed the essayist, I take the occasion to make the report conform to the nature of an essay, with more extended remarks in relation thereto.

Just at this period of the season, following a cold and protracted rain, I noted the following effects of it upon my own regiment, while stationed near Jackson, Tenn. There were about twenty-five taken off duty with the following peculiarity, in nine companies remaining in this one camp:—About ten had cold and cough, or catarrh; six took *pneumonia biliosa;* two *pneumonia typhoides;* two bronchitis with aphonia; one an intermittent, and about five with diarrhœa. In the one company stationed four miles south of Jackson, on the railroad, had nine taken sick at the same time, in which one case assumed the form of a remittent type of fever, and eight that of dysenteric flux. In this connection I draw the attention to these various cases, all arising from one cause, for two purposes; one, to show how single may be the exciting cause, and how great the variety of results under precisely the same circumstances, and, secondly, to impress the fact, that cold and wet are causes sufficiently forcible in action upon the body to induce nearly all the common diseases of camp life. I do not say that it does do it, especially during warm weather, but only speak of its efficiency during the seasons of their prevalence in combination, and upon that basis will show by what means, and through what channels, the various results are brought about.

I now propose to call attention to that part of my subject; not designing to argue the special production of any one disease, or class of diseases, but to set forth the general tendencies and common results, as applicable to any disease, which can result from sudden abstraction of heat followed by intropulsed fluids, and wish incidentally to say, that the subject has been neglected by the profession, who have been satisfied with *phenomena* alone, without being arrested by the very great importance of estimating the causation, and the paramount necessity of guarding against these agencies so common around us.

The phraseology of *taking cold* is familiar to all of us, and is even the expressive term used by literary men, derived from the common notion that they have imbibed *cold* which has found a location in the body, as it existed out of it, and like the kindred phrase of drawing out fire after a burn, has passed down into a fire side axiom.

The question, in its philosophic sense, comes up, how do we take cold? We evidently do not contract it from water, or dampness, or from the air, or from the earth, because all these elements, or partitions, of the habitable globe, are populated with birds, fishes, animals, and insects, constructed as we are, of the same constituents, of the same organic relations, and breathe the same air, drink the same water, and live upon the same food, and pass through the same process to dissolution, and yet they are not effected by it. There is nothing in air or water which, in themselves, are poisonous, or causative of disease. The whole explanation lies in the sudden abstraction of heat from the body, to the extent that *the organic forces of the economy become a deranged relation to the matter of which the body is composed.* This is effected in two ways: First, by conduction, and second, by evaporation. The process of conduction of the heat is carried on by the contiguity of damp clothing, together with the contact of air charged with floating vapor. The clothing, which when dry is porous and a poor conductor of heat, but when wet, has its porous interstices filled with watery particles, to the extent that a complete surface and uninterrupted sheet of conducting material is thus presented to carry off the heat of the body, and superadded to this silent, continuous contact, there are globules of air constantly picking up and bearing off the particles of vapor which have become warmed by the body, and substitute such as are cold. But when evaporation supercedes conduction, or both operate together, as is common, then the cooling process is still more rapid. For each globule of air feels itself especially commissioned to fill its little canteen with its 1,000° of heat, and on its pinions seek the moving gale, or the lungs of some adjacent plant or tree. In this way the air becomes very officious, at the same time, in reducing the heat of the body; for while each air particle is expanded and moving off with its caloric balloon, freighted with the warm vapor, into greater altitudes, the cold air is rushing in to fill the place, and again flies off by the expansive levity which it borrowed from the body itself.

Those medical men who have had occasion to use cold lotions to inflamed parts can comprehend, to some extent, what would be the result should they apply that same lotion to the entire body at the same time, for such is the effect of wet and cold, after a cold rain, or cold following rain. What is the result of its sudden application? It causes a sudden intropulsion of the fluids of the exterior in upon the interior organs, which of course become not only overcharged with recrementitious matter, but also of a remora of blood, which was due to the capillary surface. To compensate for this *physiological result*, (for it is yet physiological,) the interior organs are forced into a vicarious action, in order, in some way to equallize the effect and pressure. This physiological action, however, I do not array here as being any part of diseased action, but it is a normal process so excessive in extent that disease often results, not from the influx of the recrementitious matter, but from the remora of blood producing overdistension of some local capillary network. The intropulsion of the recrementitious secretion of the dermoid tissue must become of very feeble importance in the chain of abnormal phenomena, for the reason, that the secretion itself is not vitiated when it retreats from the surface, and cannot become so after the blood retreats, for the secretion is suspended until the blood returns.

If it be meant that the effete atoms of the worn out tissues are thus productive of disease, when thus suddenly driven in from the surface, I would say that the position is equally fallacious, for the same reason that the process of repletion and waste is also suspended, and there is no excess. And the idea that the changed relation of the fluids of the body from one part to another, as a cause, is of itself untenable, because the organism is in constant process of such changes, in winter and summer. That process is experienced by the diver into cold water; by the æronaut in the suddenly rarified air, also by persons descending into wells and ice-houses, during summer, etc. These physiological actions must not be looked upon as causative, but only as a normal process by which, and through which, and after which, disease does often occur.

To illustrate that point, I will introduce an engine with two boilers, with steam enough on to run a given weight in a mill or train. I connect the boilers so that they communicate with each other. I am under way, in full motion. I now bring suddenly in contact with one boiler cold enough to bring about sudden condensation of the steam back into water. The sequence of that abnormal act is this: when the condensation occurs, and a vacuum is formed, the steam forces the water from the other boiler into it until by oscillation the equilibrium is restored. But the engine stops. Why? Not because of the change of the fluids from one boiler to the other; not because of an influx or remora of fluid into the first boiler, but because of the changed relation of the *heat* to the *matter*, *water*, which propelled the engine. What was lost in this process? Not *water*, but *heat*. The *heat*, then, we observe, is the moving power, and not the water, the latter being only the recepticle, or agency, in effecting motion to other matter. Now it must not be lost sight of, that this same heat is the moving power in the animal economy, as well as everywhere in the known universe. I will state a basis of all pathological, as well as physiological action, upon which rests all the causative agency of all diseases, and it applies to all circumstances; it is this: No *diseased action can begin or exist*, except by first a *deranged relation* of the *organic forces* to the *matter of the body*, or a *changed relation of the matter of the body to the forces*. When I speak of forces, as applied to animal or vegetable, I mean heat and light, and their transitions into electricity and magnetism, and when I speak of matter, I mean the material structure of the body, whether gas, fluid, or solids. It is the heat power which effects motion, and the light, identity of place and function. I will explain this point. If any circumstances shall change the heat and light which would be required to perfect the growth of a tree, the tree would decline and die. If, on the other hand, we girdle the tree, so that the fluids are changed in their relation to the forces, light and heat, the tree also dies. Hence, it seems to me plain, that in order to have integrity of life and health of this tree, there must be the proper integrity of both the forces, and the matter. If either fails to be perfect, disease must occur, and probably death. Precisely this condition, and these circumstances, presents themselves in the vegeto-animal economy, except that both are required in greater abundance, because of greater waste. The importance of the production of these two forces may be better estimated, when it is considered that all motion of mind and body is due to one, while the replenishment is due to the other; the matter being furnished. Now when this heat of the surface was suddenly consumed, as it was in the one boiler, it became necessary to have it as suddenly restored, if possible, in order to prevent a cessation of function. Hence the heart immediately puts on the construction train, and the new material, containing a large supply of oxygen, is blindly yet vigorously furnished to the exterior, to supply the defect as soon as possible, and when the entire surface is reached, there appears an excessive action. Suddenly, instead of the pallid surface and blanched countenance, and small pulse, and dry, cold skin, and general suspension of dermoid secretion, we now have a *hot skin*, *tumid face*, *high pulse*, *hurried and suffused countenance*, the capillaries now over-distended with blood, the secretery cells overpowered with excess of the same, which still suspends secretion. This *is a condition* which has got the name of *Fever*. I will not stop here now, to treat that topic, however.

The assimilative functions, which hold their laboratory in the extreme capillaries, also suffer diminution in the same degree. Here are atoms of matter, all through the tissue, which have become effete, but not yet cast off to give place to the new matter; but, on the contrary, are yet in the cell-wall and subject to the action of oxygen, which *partially* acts upon them. Although the abnormal relations now existing between the oxygen, the blood capillaries, and absorbents will not resume function yet so much as to consume these atoms, so that they can be dissolved into fluid and be carried away; but they are oxygenated, and, while under this process, are giving out excess of heat, not to be expended in normal motions, or not to be taken up by the excretion of the skin, for it is checked, or not to be consumed by the dissolving atoms into water, for they do not dissolve; but to be spent upon *remote parts* as well as *adjacent parts*, and out of it may appear arterial excitement, wild mental action, muscular excesses, spasms, diarrhœa, cough, delirium, &c., &c., all of which are results of excessive action, evinced in the inordinate expenditure of heat; for, it must be observed, that the heat generated must be expended and compromised somewhere in the economy, and any abnormal or excessive action or motion is the evidence of excessive heat, whose expenditure has devolved upon that part for want of ready equalization. Could the worn-out atoms be liquified as fast as burned, or partly burned, the function of repletion would not be checked, and the process of food-taking in disease would not be checked by first checking the appetite; nor could there be any abnormal action when the office of waste and repletion is in balance.

The disturbance in this balance is the derangement of the matter of the body to the forces; for, when atoms of tissue are not broken down, and elimination, of course, there can be no place for new matter, and no demand. Now, this process of overheating is a reaction upon the previous abstraction of heat. This is a general process; and the true phenomena, though not always in the same degree, is as plain as the phenomena of the two boilers.

Every disturbance in the motion of either solids or fluids is caused by a disturbance in the heat force, and a derangement of the force in relation to the fluid or solid. What is the whole process of changing atoms, either in act of deposit or waste, in secretion or excretion, in production or re-production, but the new relations of matter, which, if presented to the forces always the same, must be attended with the same results, provided the forces are normal also. The process of oxygenation of the effete tissues must be either partial or complete, if acted upon at all, and in this process lies the most important to health of any physiological act in the economy. We observe, and it has escaped no physician, that the effete atoms are not removed, except in a slow process, during disease; and, in our lingering constitutional diseases, such as, for instance, typhoid fever, the oxygenation is, at first, more active and the heat higher, yet the tissues *do not change place*, and there is no call for repletion.

After a time, the oxygenation becomes more difficult,—the fever and heat declines,—still there is no change; and not until the crisis is formed, and the cell-walls give up their dead, and the old atoms begin to pass off, do we see the cheeks sink in, and emaciation become general, that the appetite arouses and repletion commences. This process of waste and repletion is the controlling function of the organism, and becomes, or is likely to be, effected by the action of cold, or by any other means by which the *forces* and the *matter* become deranged. I will not dwell longer on this most interesting topic, all of which has an especial bearing on disease.

This effect is the process, occurring wherever fever exists, or any other constitutional affection, which so generally involves the many functions of the body; and, I wish here to be understood, that the general fever does not cause this general assimilitive disturbance, but that fever is the result of it, and is *symptomatic of it.* Fever has no individuality, no more than *any other disease;* but they all result from a chain of deranged phenomena, in which the process of waste and repletion are paramount in the line of causation. Now, upon this intropulsion from sudden cold, the common laws of natural order prevails, that of *contraction* where *heat is not*, and *expansion where it is;* hence the first contractile impress is made right where colorification begins upon the surface. We now have the intropulsive; and remora takes place in capillaries of some internal organ,—say the lungs,—but, upon reaction, this remora ceases before permanent hyperemia occurs, except, probably, in a circumscribed patch of tissue in which inflammation or congestion and, finally, suppuration occurs.

Now, I ask, why did not healthy action proceed after this first engorgement, or after the hyperemia became permanent? There was no trouble now in the heat and light, for the heat has become really in excess, and just as ready to act legitimately through that spot as any other. It is this. The matter there has now become deranged, as relates to the forces. First, the forces were deranged, and now a succession of phenomena has arisen, by which the matter is deranged to the forces, and death of the part, or *local death*, has occurred.

While the effete atoms are in process of combustion and heat is being generated, the skin would soon lower the heat were its functions not arrested by the same act. More than that, the liquifying the solids is also a cooling process, for no solid can become fluid without the absorption of much heat, and in this process much of the excess of heat would be equalized, were it not for the fact, that these atoms are not thus liberated from the cell-wall or liquified. That process too is arrested; and the vicarious act of disposing of the forces is greater than that of disposing of the deranged secretion or supposed peccant humors.

As to the production of disease, it matters not whether the heat of the body is suddenly abstracted in one part, and the elements of the body inordinately taxed to reproduce it, or whether, by the reaction, occasioned by the supply of heat, has so deranged the cell function that the effete solids can be oxygenated and not liquified. In both cases, the derangement of the organic forces,—*light and heat*,— are disturbed, in relation to the matter, the same as the heat to the water in the engine. On the contrary, should I choke up one boiler with foreign matter, or contract the boiler to half size, then the engine would also stop, for I would change the relation of the matter to the force. In either case, the condition of those relations will be manifest all the way through, until the identity of the organism shall be destroyed. This brings me back to the text, that the sudden abstraction of heat by *cold and wet*, (which is the same in effect,) produces disease, either by deranging the forces to the matter or the matter the forces; and that the mere change of the fluids is not the *cause of disease*, but one process in the chain of results. Why does this process of concentrated action so often develope disease in the mucous membrane and parenchyma of the lungs? The principle reason exists in the yielding character of those tissues which have not elasticity enough to regain their lost condition from the remora. In this stage of remora the vessels become charged with blood, and eight-tenths will, upon reaction, resume their pristine level; while one-fifth will remain with distended vessels, some parts of which may run into inflammation, and others into congestion,

I would here beg leave to say, that although pus forms during a low inflammatory process, it is equally certain that the same vessel in which it forms becomes congested, or relaxed, and over-distended before the exudation period. This is the changed relation of matter to the forces. Again, the mucous membrane is more obnoxious to this intropulsion, because it is slowest in throwing off excessive influx of extraneous fluids, and slowest in its secretion and excretion. It is the most yielding tissue in the body to vascular pressure So yielding is this tissue to distension, that I doubt if true inflammation ever is developed in it, unless the adjacent tissues are equally implicated. Congestion, following remora, is, undoubtedly, the pathological condition cf the diseases which are developed in it. These two are the accumulated evidences of changed matter, in relation to the forces.

I do not mean to assert that the changed relation of forces all proceed from sudden extraction of cold. On the contrary, the act of taking cold or catarrh is often produced in dry, warm weather, and while in the house. This is owing to the electrical or magnetic condition of the air, which controls the *organic forces* of the animal system. Instances of this meterological influence upon the organic forces is shown in all the animal world, not only during disease but in health. In a word, the *forces* of the economy are directly in relation to the process of *combustion within the body*, and the forces and vicissitudes of all nature without the body; while the matter of the economy is in direct relation to the food consumed and the process of æration.

The abstraction of *heat* then, to the extent of organic derangement, first involves the *forces* by which the circulation is deranged; succeeding to this, consecutively appears in some locality, a disease in the solids, in which appears deranged matter. This diseased spot then being beyond the reparative process of repletion and waste, fails to develope the phenomena of life any longer, and must drop into other relations common to decay.

